# A rapid assessment of the National Regulatory Systems for medical products in the Southern African Development Community

**DOI:** 10.1186/s40545-020-00255-x

**Published:** 2020-10-05

**Authors:** S. Dube-Mwedzi, S. Kniazkov, J. B. Nikiema, O. M. J. Kasilo, A. Fortin, P. Tumusiime, G. N. Mahlangu, M. Ndomondo-Sigonda

**Affiliations:** 1 SADC Medicines Regulatory Harmonization Project, Harare, Zimbabwe; 2 WHO Regional Office for Africa, Health Technologies and Innovation Unit, Brazzaville, Republic of Congo; 3 Medicines Control Authority of Zimbabwe, Harare, Zimbabwe; 4 African Union Development Agency, New Partnership for Africa’s Development, Pretoria, South Africa

**Keywords:** Medical products, Medicines, Quality, Safety, Efficacy, Regulation, National Regulatory Systems, National Regulatory Authorities, Institutional frameworks, South African Development Community, UHC, SDG

## Abstract

**Background:**

Access to quality-assured, safe and efficacious medical products is fundamental for Universal Health Coverage and attaining Sustainable Development Goal 3: Ensure Healthy Lives and Well-being for All. To guarantee this right, there is a need for robust and efficiently performing national regulatory systems for the regulation of medical products. Well-functioning regulatory systems apply globally accepted standards which ensure that the level of control is proportionate to the level of public health risk.

**Objective of the study:**

The study aimed at analysing the regulatory systems for medical products in the 16 Member States of the Southern African Development Community (SADC). It provides an overview of the national regulatory systems for medical products in the region in 2017 and outlines the institutional frameworks, which enable the implementation of regulatory functions.

**Methodology:**

A survey was conducted in March-December 2017 in English, French and Portuguese. National Regulatory Authorities for medical products (NMRAs) of the 16 Member States within SADC responded to the questions asked and sent in their answers. The survey was constructed around five themes instrumental for implementation of the Universal Health Coverage actions framework. Three of the themes are discussed in this article.

**Results:**

The outcome of the survey demonstrates that within SADC, NMRAs vary in terms of organisational set-up and modalities of medical product regulation. The majority are within the Ministries of Health, and a few are either semi-autonomous or autonomous. Legal frameworks for medical products are in place for some of the SADC NMRAs, although they vary in the scope of products subject to regulation. Traditional medicines, biologicals and medical devices are regulated, however not uniformly across the region.

**Conclusion:**

Despite major progress over the years, the survey demonstrates variable levels of governance and regulatory framework among NMRAs in SADC. The survey supports the need for shifting from the broad strengthening of the regulatory systems which exist and are underpinned by the mandates, to more product-type focused approaches. This shift will ensure that medical products are quality-assured, safe and effective for a performant Health Systems attainment of the Universal Health Coverage and Sustainable Development Goals.

## Introduction

### Background

The pivotal role of health products in the global Universal Health Coverage (UHC) agenda is reflected in target 3.8 of Sustainable Development Goal (SDG) 3: Healthy lives and well-being for all at all ages. The SDG target aspires to achieve “access to safe, effective, quality and affordable essential medicines and vaccines for all” [1]. The weight of medicines vary from 6 to 45% in the national expenditures for health, yet again underlining the significant role medical products play in public health programmes [2].

Medicine regulation forms a vital link between access and quality. Well-functioning regulatory systems are necessary and underpin the quality, safety and efficacy of essential medical products, vaccines and other health technologies [3]. Such systems also pave the way for scientifically sound and cost-effective use of these products, which ultimately contributes to UHC [4].

### Universal Health Coverage (UHC)

**Fig. 1:**
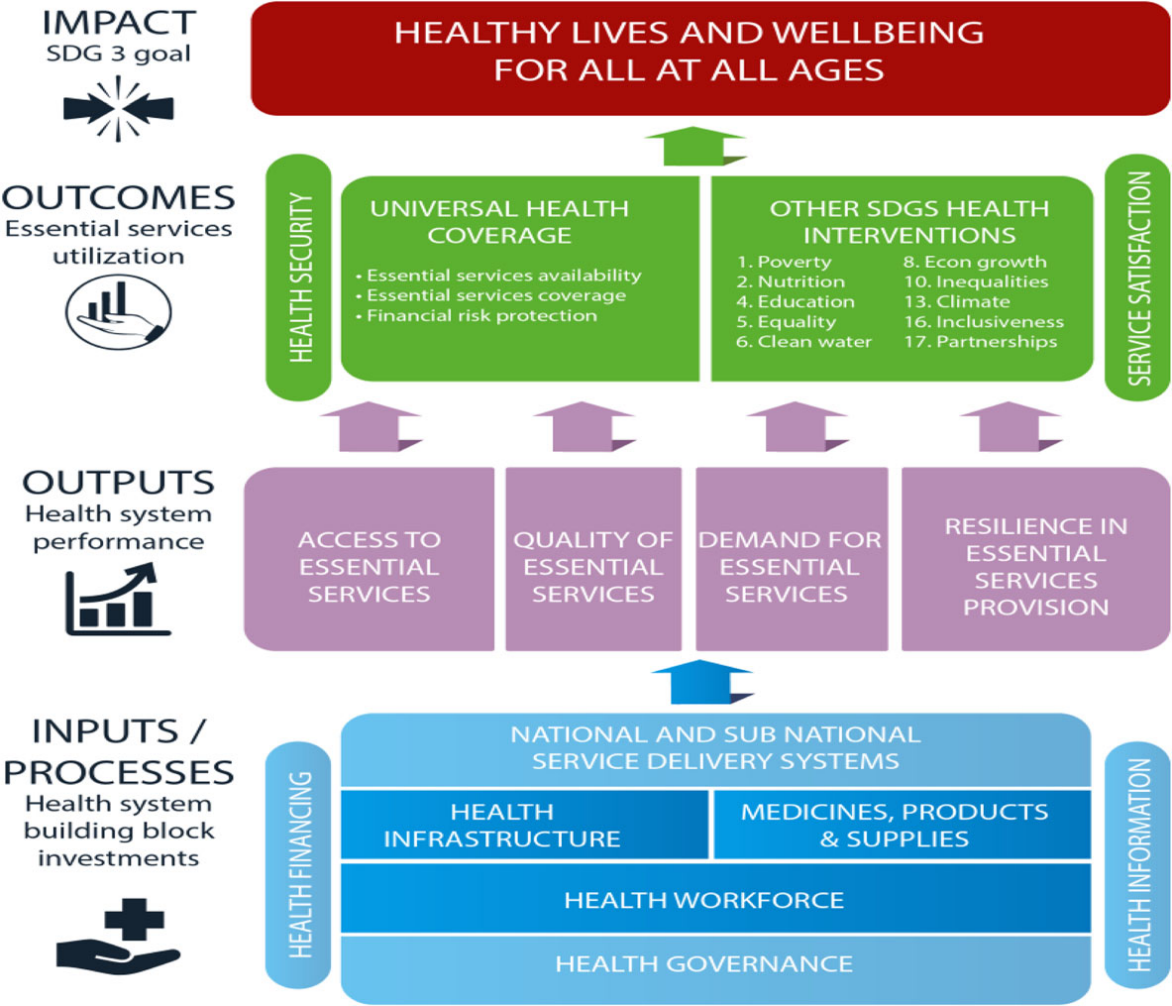
Universal Health Coverage at a Glance *Source:* WHO Regional Office for Africa, 2017

WHO defines UHC as ensuring that all people have access to needed health services (including prevention, promotion, diagnosis, treatment, rehabilitation and palliation) of a sufficient quality whilst also ensuring that the use of these services does not expose people to financial hardship [5]. Figure 1 looks at the process toward UHC attainment from the process perspective. It spells out input necessary for the achievement of UHC, helping to translate the UHC concept into action. National and subnational health delivery systems form part of the health system building block investments. Medicine regulatory systems are an integral part of these health systems. In order to guarantee the achievement of SDG 3, 80% of WHO Member States are to have optimally functioning health systems and have 80% of their population accessing these systems [6]. Assurance of achieving these targets can only be made possible by having sound medicine regulatory systems in place which are supported by robust legal frameworks, as medical products of appropriate quality are enablers and indispensable elements of effective delivery of healthcare services, as shown in Fig. 1 below [7].

On the road towards UHC, patients, communities and civil society are active contributors. Their active collaboration with the government and input of the private sector not only pave the way for UHC but also shape its characteristics: answers to the questions of what health interventions are to be included into the essential package, and the level of their coverage and (co-) contributions of the patients is no longer a prerogative of only the decision makers. In the run-up to the UN high-level meeting in September 2019, the multi-stakeholder platform UHC 2030 formulated 6 Key Asks for UHC, two of them being around product quality and enabling regulation [8]. The Lancet Commission estimated in 2018 that 60% of the global deaths per year in low- and middle-income countries are attributable to poor quality care, which surpasses the dismal toll of 3.6 million deaths annually due to poor access. Clearly, in the context of healthcare, access must be underpinned by robust regulatory systems to assure the quality of products and services [9].

### The role of medicine regulation

Every country needs an assured supply of safe, efficacious, good quality and affordable medical products to promote public health and patient care [10]. However, globally, WHO estimates that only 26% of NMRAs have adequate capacity to perform the core regulatory functions [11]. There are 47 NMRAs in the sub-Saharan Africa, but their capacity is variable with most of them non fully performing the core regulatory functions [10]. This necessitates a continued focus on regulatory systems and their strengthening, prioritisation of underperforming functions, and enforcement of regulations, which lags behind.

The mandate of NMRAs is to safeguard public health through safe and effective medical products [12]. NMRAs have an obligation to protect the health of their populations and to provide adequate mechanisms to ensure the quality, safety and efficacy of the medical products available to them throughout product life-cycle. Robust regulatory systems apply correct standards that are globally acceptable, at the same time use good risk management approaches that ensure that the level of control is proportionate to the level of public health risk [13]. To be considered robust and functional, good regulatory systems are expected to have five main characteristics: they should be responsive, outcome-oriented, predictable, risk-proportionate and independent.

### Objective of the study

**Fig. 2:**
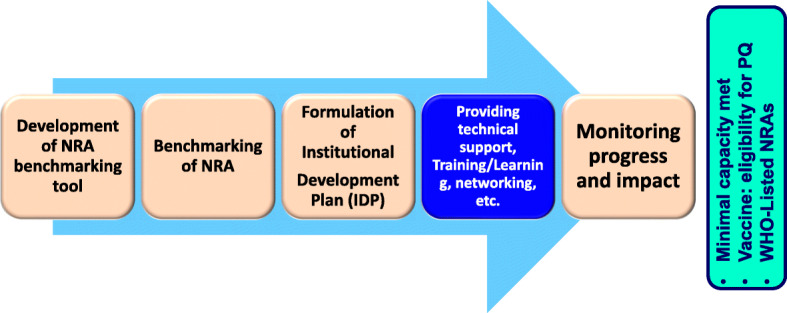
WHO five-step capacity-building model for the National Regulatory Authorities. Source: [11]

The survey was conducted by the WHO AFRO Regional Office as part of its mandate for national regulatory system strengthening, in line with World Health Assembly Resolutions including WHA67.20 endorsed by the Sixty-Seventh World Health Assembly [14]. In August 2016, the Regional Committee for Africa adopted the African Regional Strategy for Regulation of Medical Products, 2016–2025. Monitoring of the progress in regulatory systems strengthening is an essential element of the strategy [15]. This article contributes to this objective by discussing the survey findings. WHO plays a pivotal role in supporting countries in strengthening their regulatory systems and promoting equitable access to quality, safe, efficacious and affordable medicines and health products. In order to do this, WHO utilises a five-step model (Fig. 2) [16].

A baseline overview of the pharmaceutical situation within the Southern African Development Community (SADC) was undertaken in 2009. It provided an overview of demographic and economic aspects, health services data, medicine policy and access to medicines, including procurement and supply, intellectual property aspects and medicine regulation [17]. In 2010, WHO commissioned a rapid assessment of medicine regulatory systems in sub-Saharan Africa [18]. This provided further insights into the extent of medicine regulation on the continent, concluding that, although structures for medicine regulation existed, countries lacked the capacity to control the quality, safety and efficacy of the medicines in their markets or passing through their territories. In 2017, Ndomondo-Sigonda et al. [19] published an outline of the status of medicine regulation in Africa, providing an overview of the regulatory systems on the African continent, including the SADC Member States. The current article unpacks the progress in regulatory systems strengthening focussing on SADC, shedding light on the types of medical products subject to regulation, outlining regulatory functions and documenting progress towards the regulation of medical devices and diagnostics.

## Methodology

A survey was conducted in March-December 2017 among NMRAs of the 16 Member States of the SADC Region, using a self-administered, structured questionnaire. The aim of the study was to gather information that could form a basis for the review and benchmarking of regulatory systems leading to regulatory system strengthening.

The questionnaire was constructed around a model that combines the public health goal of UHC and attaining SDG 3, within the context of safeguarding public health through appropriate regulation. Five themes—governance, regulatory framework for medical products, medical devices and equipment framework, assistive technology framework and intellectual property protection, were formulated with due relevance to and accent on medical product regulation.

The questionnaire was administered in English, French and Portuguese, according to the official national language of the respondent country. Heads of NMRAs were invited to respond.

The survey was sent by email. Three e-mail messages were sent to follow-up with the recipients of the survey and to remind them about the expected deadlines to send in answers.

Responses to the survey questionnaire were received from NMRAs in twelve out of the sixteen SADC Member States, translating to a response rate of 75%. The information was collected and collated using an Excel sheet. Content analysis was done according to the themes of the questionnaire. The overview of the survey findings was presented to the heads of the NMRAs or their delegates at the 5th African Medicines Regulators Conference held on 30 November 2017 in Accra, Ghana (preliminary findings), and the SADC self-benchmarking workshops conducted on 09–13 April 2019 in Johannesburg, South Africa, and on 13–16 May 2019 in Harare, Zimbabwe, to validate the results.

The results are presented in this article as a narrative and in the form of graphs and frequencies supporting the narrative.

The information has contributed to the body of knowledge regarding regulatory systems in the SADC region and was used to demonstrate the need for the WHO assisted self-benchmarking exercise undertaken in April and May 2019. It was also used as source data for this article.

## Results

Respondents provided information on all five topics. In this article, three of the themes, namely governance, the regulatory framework for medicines and the medical devices regulation were used to demonstrate the performance of the national regulatory systems.

### Governance

**Fig. 3:**
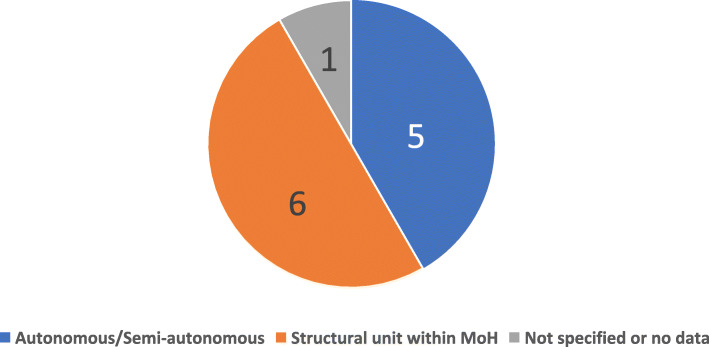
Number of countries according to the type of NMRA Governance Structure, countries within SADC (*N* = 12), 2017

As shown in Fig. 3, among the twelve respondents, six operated as a structural unit within the Ministry of Health. Five respondents were either autonomous or semi-autonomous structure. One neither confirmed the presence of a regulatory system, nor specified the type of the organisational model.

### Regulatory framework

The survey requested information on the scope of the regulatory mandate of the NMRA. It further extracted data on the life-cycle regulation scope and performance in terms of the key regulatory functions of marketing authorisation and inspection of manufacturing premises to verify compliance with Good Manufacturing Practices. A distinction was made between medicines and medical devices.

### Types of products

**Fig. 4:**
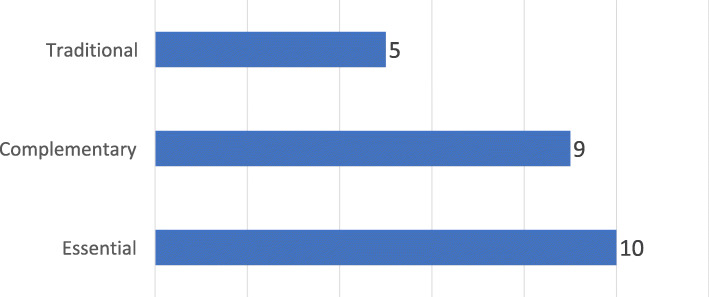
Number of countries according to the scope of Medical Product Regulation, by type of Medical Product, Countries within SADC (*N* = 12), 2017

Essential and complementary medicines constituted the greater part of the scope of product regulation (Fig. 4) for the majority of NMRAs.

Specifically, ten (10) of them included essential medicines in their scope and nine (9) included complementary medicines. As of 2017, five (5) of the responding NMRAs had systems in place for regulating traditional medicines.

**Fig. 5:**
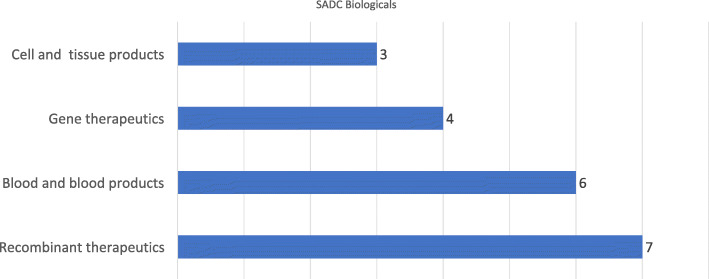
Number of countries according to the scope of biologicals regulation, by type of biological product, countries within SADC (*N* = 12), 2017

Biologicals are a relatively new class of medicines for Africa. In the survey, biologicals were broken down into the four major types and this is captured in Fig. 5.

Recombinant therapeutics and blood and blood products constituted the greater portion, for which NMRAs established regulatory processes and procedures. Only three NMRAs were regulating cell and tissue products at the time.

**Fig. 6:**
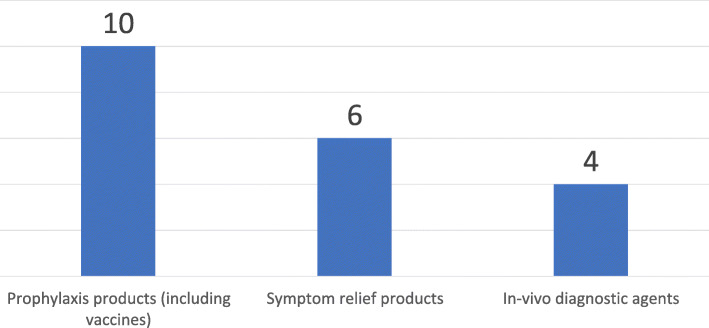
Select product types included in the definition of medicines

The definition of a medicine varied significantly among the NMRAs. Results for the functional breakdown of the definition showed the majority, ten included prophylactic products in their definition, and only six included symptom relief products. As shown in Fig. 6, only four of the NMRAs had a definition that encompassed in -vivo diagnostic agents.

### Life-cycle management

**Fig. 7:**
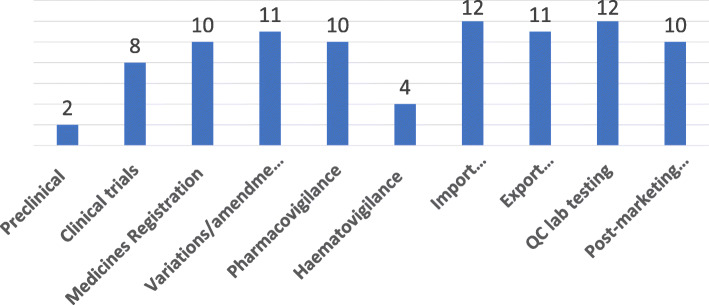
Regulatory functions throughout medicine life-cycle

Figure 7 shows the regulatory functions throughout the medicine life-cycle for the region, further breaking them down according to the survey responses. Across the continuum from pre-clinical development right up to post marketing surveillance, most of the regulation for medicines in the SADC region focused on medicine registration, pharmacovigilance, import and export control as well as quality control testing. Only two NMRAs had a framework which looked at preclinical development and only four for haemovigilance.

### Medicine registration

Each NMRA was required to provide the legal or normative references in their legislation which supported medicine regulation for both multisource (generic) and similar biological product (biosimilars). Published guidelines specific for generic medicines were available in only five, and only three of the NMRAs had published guidelines specific for biosimilars. Frequently updated registries of medical products were publicly available in five of the respondents.

The average registration time for medicines was approximately five hunderd and one (501) days. The average number of requests for medicine registration across nine NMRAs in the course of 2016–2017 was four hundred seventy-four (474). In terms of registration, the average number of products registered per NMRA in that period was two hundred eighty-nine (289).

### Good Manufacturing Practice (GMP) inspection

In 2016, eight out of the twelve responding NMRAs were conducting GMP inspections. The average number of GMP inspections was twenty-nine (29), with an average timeline of one hundred seventy-nine days (179). This timeline was taken to be the number of calendar days from the receipt of the request for inspection to the time of communication of the final outcome to the manufacturer or applicant.

Only four (4) of the responding NMRAs reported a legal framework which supported dossier evaluation and the GMP inspection process to run in parallel.

### Legal framework for medical devices and equipment

The number of NMRAs with a normative legal framework for medical devices was low, with seven (7) out of the twelve (12) respondents confirming that at least one type of the medical devices is subject to regulation.

**Fig. 8:**
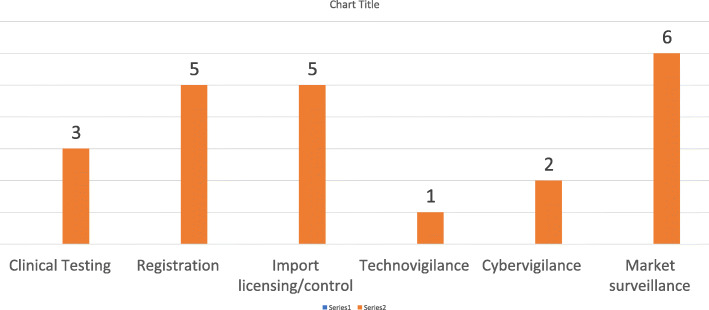
Lifecycle regulation of medical devices

Five (5) of the respondents had a legal framework for in-vitro diagnostics or imaging equipment. The regulation of medical devices mainly focused on registration and licencing (import and export) control, and to a lesser extent clinical testing and post marketing surveillance as shown in Fig. 8.

The regulation of medical products needs to be proportionate to the risks associated with their use. However, only three (3) of the respondents reported the use of medical device classifications and adjusting registration (listing) procedures.

**Fig. 9:**
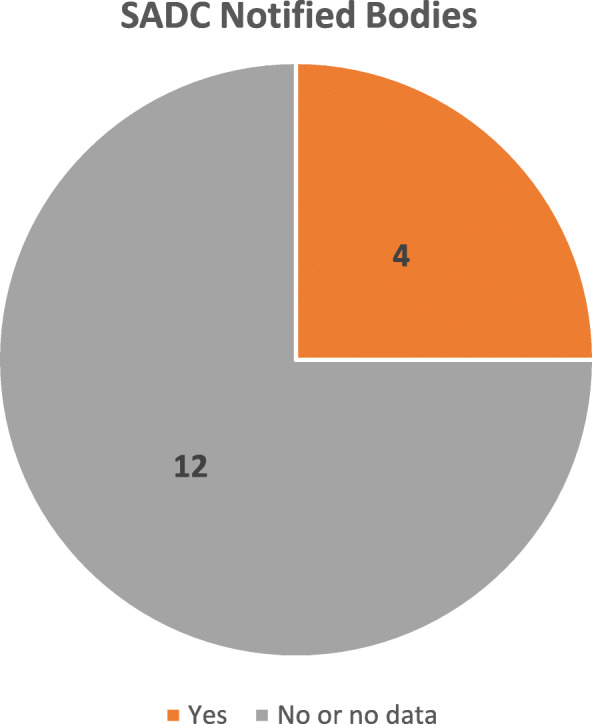
Notified bodies

Medical devices are characterised by the vast variety of their types and modifications. Specialised multi-disciplinary expertise is required to ensure appropriate oversight of their safety, quality and performance. Globally, an institute of competent (or notified) bodies exists to assist the authorities through a highly specialised expertise. As shown in Fig. 9, four (4) NMRAs in the SADC region reported reliance on the technical reports of the notified bodies in producing regulatory outcomes.

## Discussion

Success in the attainment of Universal Health Coverage is predicated by the advancement in the equally important areas: (1) financial protection, (2) access to healthcare services and medical products and assurance of their quality, safety and effiacy. The UHC and medicine regulation are intertwined—one cannot talk of one without the other. Its attainment will not be possible if the regulation is not in place and highly performant to ensure the quality of healthcare services and medical products. This means that national regulatory systems should be comprehensively functional and support the national and regional health systems with timely and predictable regulatory outcomes.

There have been structured efforts targeted at regulatory system strengthening over the years. Despite major progress in the last years, the survey demonstrates variable levels of governance and regulatory frameworks among NMRAS in SADC at the time of the study in 2017.

Complete or partial autonomy was not the predominant governance structure of the surveyed NMRAs. Not much can be concluded from such an observation, if we do not recall what impact governance has on regulatory system performance: suffice it to say that autonomy of regulatory authorities is associated with greater efficiency and effectiveness in the sense that such governance structures allow for specialisation in regulation [20], make them better responsive to healthcare needs and are perceived to be instrumental in the flexibility of resource management, which is conducive to better regulatory outcomes. Regardless of autonomy, all regulatory structures are required to have accountability mechanisms and inform the public on the key performance metrics.

Essential medicines form a pillar of healthcare globally. There are however other types of products that have emerged over time, and these requires their incorporation into the scope of regulation. In this regard, traditional medicines merit a special mention. The WHO Regional Committee for Africa adopted Resolutions AF/RC50/R3 [21] and AF/RC50/R3 [22] on promoting and enhancing the role of traditional medicine in health systems in 2000 and 2013, respectively. As of 2017, 32% of the responding NMRAs had systems in place for regulating traditional medicines, demonstrating a certain level of political will in regulating this category of medicines that are widely used in most countries around the world and whose uptake is increasing [23].

The extent of incorporation of regulatory frameworks for biologicals was low in the SADC region. This could be attributed to a lack of technical capacity in regulating these products. Whilst the region seemed to have made acceptable progress with respect to the traditional regulatory functions and processes, the life-cycle regulation study highlighted areas where system strengthening can focus. There needs to be a focus on areas such as preclinical development, clinical trials and product-specific vigilance as the region is supported to embrace lifecycle regulation.

The availability of information in the public domain is pertinent to both the regulator and the regulatee. Public availability of legal or normative references reported by only half of the respondents should be a cause for concern for the region. There is a need to support the region to take advantage of the digital age, and the role it places in information dissemination. Similarly, the extent of public awareness of and access to registries needs to be improved.

A robust regulatory system focuses on regulatory outcomes whilst being equally concerned about processes. It should also fulfil the desired characteristic of being predictable. Registration timelines are used as one of the metrics of the efficiency of NMRAs. The European Medicines Agency (EMA) and United States Food and Drug Authority (US FDA), both of whom are proposed to be grand-fathered as WHO- Listed Authorities , have comparable average timelines for medicine registration, which are 369 days and 303 days, respectively [24]. The longer timeline averaging approximately 500 days for the SADC region could be attributed to technical capacity issues or high staff turnover. Furthermore, these timelines need to be viewed in the context of limited resources. As some NMRAs within the region gain technical strength, there is a possibility of robust registration procedures, which require a comprehensive in-depth review of causes for  protracted timelines.

Granting or rejecting marketing authorisation is based on a combined consideration of the dossier assessment outcome and the GMP inspection. Concurrent conduct of these two processes could speed up the final outcome and make medicines accessible to patients faster. The number of NMRAs having this in place was moderate, applying to seven of the twelve respondents.

For many years, efforts have been focused on strengthening regulatory frameworks for medicines, whilst focus on medical devices and in vitro diagnostics (IVDs) has not been equally high. Yet, poor quality medical devices and in vitro diagnostics have a major impact on healthcare outcomes and, ultimately, on people's health. It is widely accepted now that the value of medication rests largely on the accuracy of the diagnosis, and thus, the use of poor quality medical devices and diagnostics undermines the effectiveness of efforts related to making good quality, safe and efficacious medicines available. Investing more efforts into strengthening frameworks for medical devices could be recommended as a priority for the SADC countries.

Risk-based classification of medical devices allows the region to focus efforts on products with higher hazard probability, something that was lacking at the time of the study.

It is important to exercise appropriate control over the notified bodies. However, this could be the subject of a more specialised overview.

### Recommendations

The regulatory systems landscape in SADC has not been stagnant over the years. It is apparent from the 2017 data that there are differing levels of expertise and frameworks. The core functions of NMRAs need to be expanded across the region, and in particular, to include preclinical development, product-specific expertise in the product life-cycle regulation scope. It is also critical to bridge the gaps by making the mandate of the regulatory authorities comprehensive to encompass all types of medical products (e.g., broaden it to include traditional and complementary products, in vivo diagnostic agents, make provisions for medical devices and the emerging framework of assistive technologies). The options for formulating policy priorities in the SADC region could include a specific focus on certain types of products (e.g., blood, medical devices and in vitro diagnostics) or expansion of regulatory functionality into new types of products (e.g., biologicals).

The overview reveals a need to transition from the broad strengthening of the regulatory systems, which exist and are underpinned by the mandate in the majority of the NMRAs, to more product type-focussed approaches. This will entail both a wider perspective in terms of the scope and type of product, as well as specialisation of the regulatory functions to, for example, include haemovigilance and other targeted forms of pharmacovigilance.

## Conclusion

There is a need to continue strengthening NMRAs within the region to ensure both achieving SDG3 by 2030 and the objectives of the Regional Strategy for Regulation of Medical Products, 2016–2025; and assuring the quality, safety and efficacy of available medical products.

The efficiency and effectiveness of regulatory systems are beginning to take a central stage, and regulators must be encouraged to invest in gauging their performance and information sharing. Responsiveness is a component of measuring robustness. Regulation needs to move in tandem with the changes becoming apparent in medical and pharmaceutical practice. A case in point is that medicine availability is no longer restricted to brick-and-mortar structures, necessitating a serious look into cyber space.

There is little or no documented evidence of the extent of medical product regulation in the region.

## Data Availability

The datasets used and analysed during the study that are the main basis for the review are available from the corresponding author on request.

## Abbreviations

EMA: European Medicines Agency
GMP: Good Manufacturing Practices
IVD: In vitro diagnostic
NMRAs: National Medicines Regulatory Authorities
PDR: Prevention, Detection and Response
SADC: Southern African Development Community
SDG: Sustainable Development Goal
UHC: Universal Health Coverage
USFDA: United States Food and Drug Authority
WHO: World Health Organization
